# Rethinking pN1 Disease in Non-Small Cell Lung Cancer: Anatomical Subclassification, Surgical Extent, and Survival Outcomes

**DOI:** 10.3390/jcm15103950

**Published:** 2026-05-20

**Authors:** Eyüp Halit Yardımcı, Aleyna Gültekin Arıdaş, Sezer Aslan, Tunç Laçin, Korkut Bostancı

**Affiliations:** Thoracic Surgery Department, School of Medicine, Marmara University, 34854 Istanbul, Turkey; gltekinaleyna@gmail.com (A.G.A.); sezer.aslan@marmara.edu.tr (S.A.); tunc.lacin@marmara.edu.tr (T.L.); kbostanci@marmara.edu.tr (K.B.)

**Keywords:** non-small cell lung cancer, lymph node metastasis, pN1 subclassification, TNM staging, extent of resection, survival analysis

## Abstract

**Background**: Pathological N1 (pN1) non-small cell lung cancer (NSCLC) presents variable survival; yet, the TNM system lacks N1 subclassification. While studies focus on numerical nodal burden, the prognostic impact of anatomical location remains unclear. Surgically, completion lobectomy is advised after sublobar resection for N1-positive disease. However, for hilar/interlobar involvement—where residual lymphatic pathways remain post-lobectomy—extension to pneumonectomy is rarely performed, raising uncertainty about the optimal extent of resection in different pN1 subgroups. **Methods**: This retrospective study evaluated 150 patients with pN1 NSCLC who underwent curative-intent anatomical lung resection and systematic nodal dissection (2012–2023). The follow-up period extended from the date of surgery to death or last follow-up, with survival status assessed until March 2026. Clinicopathological variables, including anatomical N1 level, nodal burden, tumor characteristics, and surgical extent, were analyzed alongside survival outcomes. **Results**: Peripheral N1 involvement (stations 12–14) yielded significantly longer survival than hilar/interlobar metastasis (stations 10–11) (*p* = 0.019). Nodal count and multiple-station involvement did not impact survival. Age (HR: 1.036, *p* = 0.026) and interlobar station 11 pN1 positivity (HR: 1.912, *p* = 0.044) emerged as independent negative prognostic factors for overall survival. Perineural invasion worsened survival in Stage III disease. Extended resections offered no survival benefit and worsened outcomes in hilar/interlobar disease. **Conclusions**: The anatomical level of N1 metastasis is a key prognostic factor in pN1 NSCLC. Standard lobectomy appears sufficient across all subgroups, including hilar/interlobar disease, while extended resections do not improve survival. Future studies should clarify systemic/adjuvant treatment strategies.

## 1. Introduction

Surgical resection remains the primary treatment modality for early-stage non-small cell lung cancer (NSCLC). Regional lymph node (N) involvement is one of the most critical factors determining postoperative survival. The current 9th TNM staging system, based on data from the International Association for the Study of Lung Cancer (IASLC), has improved prognostic accuracy by subdividing N2 metastases into single-station (N2a) and multiple-station (N2b) involvement [1]. However, a similar anatomical or numerical subclassification has not yet been established for N1 disease. N1 metastasis represents a highly heterogeneous patient cohort with survival rates spanning a wide range [2]. In the current staging system, hilar (station 10), interlobar (station 11), or more peripheral (stations 12–14) involvement, as well as single versus multiple-station N1 positivity, are not reflected in the staging and are evaluated within the same risk category. This lack of granularity may obscure clinically meaningful differences in tumor biology and patterns of lymphatic dissemination within the N1 category, and may also have implications beyond prognostication, as more precise identification of high-risk pN1 subgroups could influence postoperative risk stratification and future decisions regarding adjuvant systemic treatment. Therefore, anatomical subclassification may be clinically relevant not only for surgical planning but also for tailoring multidisciplinary postoperative management. In particular, the anatomical progression of lymphatic spread suggests that not all N1 involvement carries the same biological and prognostic significance. Therefore, treating N1 disease as a uniform entity may limit the ability of current staging systems to accurately stratify risk and guide treatment decisions.

The indication for surgical treatment in patients with N1 positivity is well-established; however, the extent of resection remains an area of investigation. Recent large-scale trials, such as JCOG0802 and CALGB 140503, have clearly demonstrated that sublobar resections, such as segmentectomy, are non-inferior to lobectomy in terms of oncological outcomes and survival for peripheral early-stage NSCLC [3,4]. Despite this, the generally accepted approach for patients found to have intraoperative or postoperative N1 positivity is that sublobar resection is inadequate and the surgery should be completed to a lobectomy.

However, this approach harbors a conceptual contradiction within our ongoing surgical practices. In a patient undergoing a standard lobectomy where hilar or interlobar lymph nodes are found to be positive upon pathological examination of the specimen, the procedure is not routinely extended to a larger resection or pneumonectomy merely to fulfill oncological surgical principles. While highly morbid pneumonectomy is avoided in cases of station 10–11 positivity following lobectomy, the belief that surgery must absolutely be completed to a lobectomy in the presence of more peripheral N1 positivity following sublobar resection is open to debate. This paradox stems from the fact that the prognostic value of N1 subgroups is not yet fully understood.

Although some studies in the literature highlight the survival difference between single-station and multiple-station N1 positivity [5], there is no definitive consensus regarding the prognostic value of the number of metastatic lymph nodes and the N1 station level (hilar, interlobar vs. peripheral). This limitation has contributed to the ongoing uncertainty regarding whether differences in survival are driven by the extent of nodal involvement, the location of metastasis, or a combination of both. Furthermore, data on the impact of additional pathological features of the primary tumor—such as pleural invasion (PLI), lymphovascular invasion (LVI), and perineural invasion (PNI)—combined with the existing N1 burden (number of involved N1 stations and the total number of metastatic N1 lymph nodes), on survival in pN1 cases are quite limited [6,7]. Consequently, a more comprehensive evaluation incorporating both anatomical and pathological parameters is warranted.

The objective of this study is to evaluate the factors associated with a poor postoperative prognosis in surgically treated pN1 NSCLC cases. By investigating the impact of the metastatic N1 station level, the number of involved stations, the number of metastatic lymph nodes, and the additional pathological features of the primary tumor on survival, we aim to determine which pN1 subgroups are associated with a worse prognosis and to examine the contribution of the extent of surgery to survival, specifically based on the metastatic station.

## 2. Materials and Methods

### 2.1. Study Design and Patient Selection

This study was designed as a retrospective, single-center cohort analysis conducted at a high-volume tertiary thoracic surgery institution. Institutional electronic medical records, operative reports, and pathology databases were systematically reviewed to identify patients who underwent curative-intent anatomical lung resection for NSCLC between January 2012 and December 2023. The study protocol adhered to the principles outlined in the Declaration of Helsinki, and institutional review board approval was obtained prior to data collection. The requirement for individual informed consent was waived by the ethics committee due to the retrospective design and complete anonymization of patient data.

Eligible patients were those with a final pathological diagnosis of pN1 disease following complete (R0) anatomical resection, defined as at least lobectomy with systematic mediastinal lymph node dissection. To minimize confounding related to treatment-induced nodal downstaging or sterilization, only patients who underwent upfront surgical resection without neoadjuvant chemotherapy or radiotherapy were included. Pediatric patients were excluded.

A total of 169 patients initially met the inclusion criteria. Nineteen patients were excluded due to the following reasons: perioperative mortality within 30 days of surgery (n = 4), incomplete resection (R1/R2) (n = 12), histological diagnosis of small cell lung carcinoma (n = 2), and typical carcinoid tumor (n = 1). The final study cohort consisted of 150 patients with pathologically confirmed pN1 NSCLC.

### 2.2. Pathological Evaluation and Nodal Classification

All surgical specimens were processed according to standardized pathological protocols. Tumors were classified histologically in accordance with the World Health Organization classification of lung tumors. Tumor size, T descriptor, and overall pathological stage were determined based on the current TNM classification system.

Lymph node stations were defined according to the IASLC [8]. Station 10 (hilar) nodes were defined as lymph nodes immediately adjacent to the mainstem bronchus and pulmonary vessels at the lung hilum. Station 11 (interlobar) nodes were located between the lobar bronchi within the interlobar fissures. More peripheral stations included station 12 (lobar nodes), located along the lobar bronchi; station 13 (segmental nodes), situated along the segmental bronchi; and station 14 (subsegmental nodes), found along the subsegmental bronchi within the lung parenchyma.

For the purposes of this study, N1 lymph node involvement was anatomically subclassified into two groups: hilar/interlobar (stations 10–11) and peripheral (stations 12–14). In patients with metastases involving multiple N1 stations, classification was based on the highest (most proximal) involved station, reflecting the presumed stepwise biological progression of lymphatic spread toward the mediastinum.

In addition to nodal status, pathological features of the primary tumor—including LVI, VPI, and PNI—were recorded, as these factors have been implicated in tumor aggressiveness and recurrence risk [6,7].

### 2.3. Preoperative Evaluation and Surgical Procedure

All patients underwent a standardized preoperative staging protocol, including whole-body positron emission tomography-computed tomography (PET-CT) and contrast-enhanced cranial magnetic resonance imaging (MRI), to exclude distant metastases and confirm clinical M0 status. Invasive mediastinal staging procedures were performed selectively based on radiological suspicion, in accordance with contemporary clinical guidelines.

The choice of surgical approach (open thoracotomy versus minimally invasive techniques) was determined by surgeon preference and tumor characteristics. Systematic nodal dissection was intended to be performed in all cases in accordance with the European Society of Thoracic Surgeons (ESTS) guidelines, irrespective of surgical approach [9]. Mediastinal lymph node dissection included the subcarinal station, at least one upper and one lower mediastinal station, as well as the hilar and interlobar nodes in all patients. All surgical procedures were performed with strict adherence to standard oncological principles, ensuring complete resection and systematic nodal dissection in every case. Consequently, both open and minimally invasive procedures were performed according to the same oncological principles, with the aim of achieving complete resection and systematic nodal evaluation.

The extent of pulmonary resection was categorized into two groups based on surgical magnitude: standard lobectomy and extended resections. Extended resections comprised bilobectomy, sleeve lobectomy, and pneumonectomy. The decision to perform extended resection was primarily based on tumor size, centrality, and local invasiveness, rather than nodal involvement alone.

### 2.4. Data Collection and Outcome Measures

Demographic, clinical, surgical, and pathological data were extracted from institutional databases. Variables collected included age, sex, tumor histology, tumor size (T descriptor), overall TNM stage, tumor location, type and side of resection, number and location of metastatic lymph nodes, and presence of LVI, VPI, and PNI.

Postoperative follow-up was conducted according to institutional protocols, typically involving clinical evaluation and thoracic imaging at regular intervals (every 3–6 months during the first two years and annually thereafter). Survival status was verified through hospital records and, when necessary, national registry systems. The end of the follow-up period for survival analysis was defined as March 2026. The primary outcome measure was overall survival (OS), defined as the time from the date of surgery to death from any cause or last follow-up.

### 2.5. Study Objectives and Subgroup Definitions

The study was structured around three primary analytical objectives. First, the prognostic impact of N1 anatomical location was evaluated by comparing survival outcomes between patients with peripheral (stations 12–14) and hilar/interlobar (stations 10–11) nodal involvement. Additionally, the effects of multiple-station involvement and total metastatic lymph node count were assessed.

Second, the relationship between the extent of pulmonary resection and survival was examined within each N1 anatomical subgroup. Patients were stratified into standard lobectomy and extended resection groups, and survival outcomes were compared accordingly.

Third, the prognostic significance of tumor-related pathological features (LVI, VPI, and PNI) was analyzed within stage-specific subgroups (Stage II vs. Stage III), to evaluate their interaction with nodal disease burden.

### 2.6. Statistical Analysis

Categorical variables were presented as frequencies and percentages, whereas continuous variables were expressed as medians and ranges (or interquartile ranges). The normality of continuous data distribution was assessed using the Kolmogorov–Smirnov test. Given that the continuous variables were not normally distributed, non-parametric tests were utilized. The Mann–Whitney U test was used to compare differences between two independent groups, while the Kruskal–Wallis test was applied for comparisons involving three or more groups. Correlations between variables were evaluated using Spearman’s rank correlation analysis.

Survival curves were estimated using the Kaplan–Meier method, and differences in survival between groups were compared using the log-rank test. To identify independent prognostic factors affecting survival, multivariable analysis was performed using Cox proportional hazards regression models. Variables that demonstrated a statistically significant correlation in the univariate analysis were included in the multivariable regression model as dummy variables.

All statistical analyses were conducted using IBM SPSS Statistics for Windows, version 25.0 (IBM Corp., Armonk, NY, USA). A two-sided *p*-value of <0.05 was considered to indicate statistical significance, and 95% confidence intervals (CIs) were reported.

## 3. Results

### 3.1. Baseline Characteristics and Survival Status

A total of 150 patients with pN1 NSCLC were included in the study. At the end of the follow-up period, 72 patients (48.0%) had died (non-survivor group), while 78 patients (52.0%) remained alive (survivor group). The survivor versus non-survivor comparison was performed as an initial descriptive overview of the overall cohort according to final survival status and was not intended to define the primary grouping methodology of the study. The primary analytical grouping was based on anatomical pN1 location, whereas surgical extent was evaluated as a secondary grouping variable.

When the distribution of clinicopathological characteristics was analyzed between the two groups, the overall TNM stage differed significantly (*p* = 0.026). Specifically, Stage IIA was more frequently observed in the survivor group, whereas Stage IIIA was significantly more common among the non-survivors. Furthermore, the presence of VPI was found to be significantly higher in the non-survivor group (*p* = 0.029).

Conversely, there were no statistically significant differences between the survivor and non-survivor groups regarding gender, age (*p* = 0.051), T descriptor (*p* = 0.076) tumor histology, type of pulmonary resection, side of operation, lobar location, the specific level of positive pN1 stations (hilar/interlobar vs. peripheral), the total number of positive lymph nodes, the presence of multiple-station pN1 disease, LVI, and PNI (*p* > 0.05 for all) (Table 1).

### 3.2. Univariate and Multivariable Survival Analysis

Univariate correlation analysis revealed that survival duration was significantly and negatively correlated with increasing age (r = −0.237, *p* < 0.01), T descriptor (r = −0.253, *p* < 0.01), overall TNM stage (r = −0.213, *p* < 0.01), left-sided operations (r = −0.190, *p* < 0.05), the presence of PNI (r = −0.164, *p* < 0.05), and the presence of VPI (r = −0.257, *p* < 0.01) other than positive pN1 station status (Table 2).

In the multivariable Cox proportional hazards regression model incorporating significant variables from the univariate analysis, age and interlobar station 11 pN1 positivity remained independent negative prognostic factors for overall survival. Increasing age was associated with poorer survival (HR: 1.036, 95% CI: 1.004–1.068, *p* = 0.026), and interlobar station 11 pN1 positivity was also independently associated with worse overall survival (HR: 1.912, 95% CI: 1.018–3.593, *p* = 0.044). The effects of T descriptor, TNM stage, PNI, and VPI did not reach statistical significance in the multivariable model (Table 3).

After the overall descriptive and multivariable survival analyses, baseline clinicopathological characteristics were compared according to the primary anatomical pN1 grouping of the study: peripheral pN1 disease, defined as involvement limited to stations 12–14, and hilar/interlobar pN1 disease, defined as involvement of stations 10–11. Adenocarcinoma was more common in the hilar/interlobar pN1 group, whereas squamous cell carcinoma was more common in the peripheral pN1 group (*p* = 0.010). Multiple-station pN1 involvement was also significantly more frequent in the hilar/interlobar pN1 group (*p* < 0.000). No statistically significant differences were observed between the two anatomical pN1 groups in terms of age, TNM stage, type of pulmonary resection, surgical approach, number of positive lymph nodes, lymphovascular invasion, perineural invasion, or visceral pleural invasion (Table 4).

### 3.3. Impact of N1 Station Location on Survival

**Figure 1:**
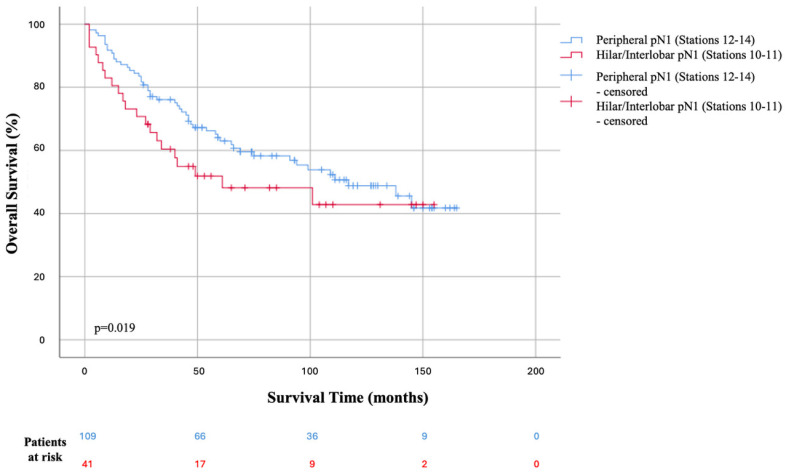
Kaplan–Meier curves for overall survival stratified by the anatomical level of pN1 lymph node metastasis.

When survival times were evaluated according to the anatomical level of the N1 metastasis, a statistically significant difference was observed. The mean survival time for patients without hilar/interlobar (stations 10 or 11) involvement (i.e., those with only peripheral station 12–14 involvement) (number of patients “n” = 109) was significantly higher than that of patients with station 10 or 11 positivity (n = 41) (73.61 ± 47.79 months vs. 54.44 ± 45.65 months, *p* = 0.019) (Table 5). Overall survival analysis similarly demonstrated that the estimated mean overall survival was 102.41 ± 6.38 months for patients without station 10/11 involvement (peripheral pN1), compared to 83.80 ± 10.74 months for those with station 10/11 positivity, confirming a statistically significant survival advantage for the peripheral pN1 group (*p* < 0.05) (Figure 1).

Patients with hilar/interlobar involvement (stations 10–11, red line) demonstrated a significantly worse overall survival compared to those with strictly peripheral pN1 disease (stations 12–14, blue line) (*p* = 0.019, log-rank test). Vertical cross marks (+, censored) indicate patients who were alive at the end of the follow-up period.

### 3.4. Surgical Extent and N1 Station Interaction

**Figure 2:**
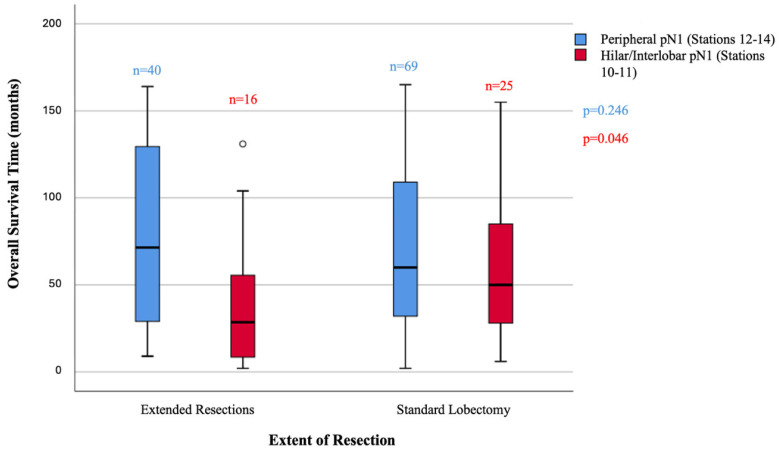
Comparison of overall survival time by the extent of surgical resection within pN1 anatomical subgroups. Box-plots representing overall survival time stratified by the surgical approach and pN1 anatomical subgroups. The x-axis denotes the extent of pulmonary resection (extended resections vs. standard lobectomy). Within each surgical category, the blue boxes (left) represent patients with strictly peripheral pN1 involvement (stations 12–14), while the red boxes (right) represent patients with hilar or interlobar pN1 involvement (stations 10–11). The boxes indicate the median, 25th, and 75th percentiles; whiskers represent the non-outlier range; circles (o) indicate outliers. “n” stands for number of patients above each plot. No significant survival difference was observed within the peripheral pN1 group (*p* = 0.246), whereas in the hilar/interlobar pN1 group, standard lobectomy provided a significantly better median survival compared to extended resections (*p* = 0.046).

The impact of the extent of pulmonary resection on survival was further analyzed within the N1 station subgroups. In patients without station 10 or 11 involvement (peripheral pN1), the survival time did not differ significantly between standard lobectomy (“lesser” resection) (n = 69) and extended resections (pneumonectomy, sleeve resection, or bilobectomy) (n = 40) (68.87 ± 44.70 months vs. 81.80 ± 52.26 months, respectively; *p* = 0.246) (Table 6, Figure 2). Conversely, in the cohort with hilar/interlobar (station 10 or 11) involvement, patients who underwent standard lobectomy (n = 25) had a significantly longer mean survival time compared to those who underwent extended resections (n = 16) (63.80 ± 47.06 months vs. 39.81 ± 40.48 months, *p* = 0.046) (Table 6, Figure 2).

Extended resections include pneumonectomy, bilobectomy, and sleeve lobectomy. These findings should be interpreted cautiously because extended resections were more likely to be performed for larger, more central, or locally invasive tumors.

### 3.5. Prognostic Impact of Tumor Characteristics Based on Overall TNM Stage

Further subgroup analysis was performed by stratifying the cohort according to the overall TNM stage (Stage II vs. Stage III) to evaluate the impact of multiple-station N1 disease and additional tumor characteristics (LVI, PNI, VPI). While these variables did not show a statistically significant effect on survival within the Stage II (IIA and IIB) (n = 80) subgroup (*p* > 0.05 for all), the presence of PNI emerged as a significant negative prognostic factor in Stage III patients (n = 70). Specifically, within the Stage III cohort, the mean survival time for patients with PNI was significantly shorter than for those without PNI (38.16 ± 36.30 months vs. 66.67 ± 49.51 months, *p* = 0.027) (Table 7).

## 4. Discussion

Although surgical resection is the cornerstone of curative-intent treatment for NSCLC, the presence of pN1 metastasis introduces significant prognostic variability that current staging systems fail to fully address. In this retrospective cohort, we sought to clarify the prognostic significance of specific N1 nodal stations and their interplay with the extent of surgical resection. Our findings confirm that pN1 is not a uniform disease state; patients with strictly peripheral nodal metastasis (stations 12–14) exhibited significantly prolonged survival compared to those with hilar or interlobar involvement (stations 10–11). Importantly, our data indicate that standard lobectomy provides sufficient oncological clearance and remains the potentially adequate surgical approach for this patient population. Given the inherent physiological burden and higher morbidity associated with extended resections, increasing extent of resection did not yield a survival benefit in peripheral pN1 disease, and conversely, resulted in poorer overall survival in patients with proximal (hilar/interlobar) nodal involvement.

While the current TNM classification groups all N1 metastases into a single category, emerging evidence from various international and regional cohorts suggests that the anatomical level of the involved lymph nodes significantly dictates survival [5,10,11]. Consistent with these observations, our cohort demonstrated that patients without station 10 or 11 involvement had a markedly better survival time (73.61 vs. 54.44 months, *p* = 0.019). It is postulated that hilar and interlobar lymph nodes represent a more advanced step in the lymphatic drainage pathway, serving as a gateway to the mediastinum (especially hilar) due to their anatomical location, and thus exhibit a biological behavior closer to N2 disease [12,13,14]. The higher incidence of poor outcomes in station 10 and 11 disease can be attributed to their direct and robust lymphatic connections to the mediastinal compartments, allowing cancer cells to bypass regional barriers [12,13,14,15].

The necessity of extended resections versus standard approaches to achieve complete oncological clearance remains highly debated [4,7]. For strictly peripheral N1 disease (stations 12–14), extending the resection beyond a standard lobectomy yielded no additional survival advantage (81.80 vs. 68.87 months, *p* = 0.246), supporting the adequacy of lobectomy in this subgroup. Conversely, for hilar/interlobar N1 disease (stations 10–11), patients who underwent standard lobectomy survived significantly longer than those subjected to extended resections, such as pneumonectomy or sleeve resections (63.80 vs. 39.81 months, *p* = 0.046). However, this finding should be interpreted cautiously. Extended resections were not randomly assigned and were generally performed in patients with more central, larger, or locally invasive tumors. Therefore, the inferior survival observed in the extended resection group may reflect not only the physiological burden of more extensive surgery but also underlying tumor biology and anatomical complexity. Accordingly, our results should not be interpreted as definitive evidence that standard lobectomy is superior in all patients with hilar/interlobar pN1 disease, but rather as evidence that escalation of resection solely based on proximal N1 involvement is not supported by the present data. This inverse relationship may be partly attributable to the higher physiological burden, potential morbidity, diminished postoperative cardiopulmonary reserve, and more aggressive tumor characteristics associated with cases requiring extended resections, which may offset any theoretical oncological benefit of wider margin clearance [5,9,16]. Therefore, pushing the surgical limits solely to achieve complete nodal clearance in proximal station positive cases appears unwarranted.

The recent proposals by the IASLC for the forthcoming 9th edition of the TNM classification considered subclassifying pN1 disease based on the number of involved stations, defining N1a as single-station and N1b as multiple-station metastasis [1]. However, in our cohort, multiple-station positivity did not emerge as a statistically significant prognostic factor for survival when compared to single-station disease (*p* > 0.05), even the frequency of having multiple positive stations was higher in the hilar/interlobar pN1 group. Instead, the anatomical level of the involved lymph nodes proved to be a far more critical determinant of patient outcomes. Based on our findings, we argue that simply counting the number of metastatic stations is insufficient for accurate prognostication. A more precise and clinically relevant subclassification for future TNM staging systems would be to stratify N1 disease anatomically: defining N1a as strictly peripheral metastasis (stations 12–14) and N1b as hilar/interlobar metastasis (stations 10–11). This anatomical division not only better reflects the underlying tumor biology and lymphatic drainage pathways [2,14], but also serves as a clinically relevant prognostic indicator that may inform the optimal extent of surgical resection.

The lack of complete adjuvant chemotherapy data is particularly relevant when interpreting survival differences between anatomical pN1 subgroups. Because pN1 disease generally represents an indication for postoperative systemic therapy, differences in the administration, completion, or tolerance of adjuvant treatment may have influenced overall survival. This limitation may be especially important for patients with hilar/interlobar pN1 involvement, in whom more proximal nodal spread may reflect a higher-risk biological phenotype and in whom systemic therapy could potentially modify the adverse prognostic effect of nodal location. Therefore, the observed survival disadvantage in the hilar/interlobar group may reflect not only nodal anatomy and tumor biology but also unmeasured differences in adjuvant treatment exposure.

Numerous studies suggest that multiple-station N1 involvement and high lymph node burden negatively impact survival, even in early-stage disease [17,18]. However, when stratifying our patients by overall TNM stage, multiple-station N1 involvement was not a statistically significant predictor of poor prognosis in either early-stage (Stage II, *p* = 0.438) or advanced-stage (Stage III, *p* = 0.879) disease. Similarly, tumor-related factors such as VPI and LVI, which are often associated with an increased risk of recurrence [6], did not emerge as distinguishing prognostic factors in our early-stage cohort. Among the tumor invasion parameters, PNI was the only factor that exerted a significant impact on survival, and this effect was observed exclusively in Stage III patients. Within the Stage III cohort, the presence of PNI reduced the mean survival time from 66.67 months to 38.16 months (*p* = 0.027). PNI is generally considered a marker of aggressive tumor biology [7]. In our study, PNI did not significantly affect survival in Stage II disease, whereas it was associated with markedly shorter survival in Stage III patients. This may suggest that the adverse prognostic effect of PNI becomes more clinically apparent in the presence of a higher overall tumor burden. In Stage III disease, where larger tumor size, more advanced local invasion, or more complex nodal spread may already exist, PNI may act as an additional biological marker identifying a subgroup with particularly aggressive behavior. However, because recurrence data and adjuvant treatment details were incomplete, the relationship between PNI, recurrence patterns, and treatment response could not be fully evaluated.

Taken together, these findings highlight that the prognostic landscape of pN1 NSCLC is more complex than currently reflected in existing staging frameworks. Rather than representing a homogeneous intermediate category, pN1 disease appears to encompass biologically distinct subgroups defined by the anatomical level of nodal involvement. This distinction has direct implications not only for prognostication but also for surgical strategy and overall treatment planning. Importantly, our results suggest that increasing the extent of pulmonary resection does not compensate for the adverse biology associated with more proximal nodal spread, underscoring the need to move beyond purely anatomy-based surgical escalation. Instead, a more refined approach that integrates nodal anatomy with tumor biology may provide a more rational basis for clinical decision-making. In this context, anatomical subclassification of N1 disease may serve as a practical and immediately applicable tool to better stratify patients and optimize treatment strategies without introducing additional complexity to current clinical workflows.

### Limitations

Our study has several limitations that should be acknowledged, primarily inherent to its retrospective design. Apart from the single-center design and relatively limited sample size, first, due to incomplete long-term follow-up records regarding disease recurrence across the cohort, our analysis was restricted to overall survival, and disease-free survival (DFS) could not be evaluated. The absence of DFS data also limits the interpretation of the relationship between surgical magnitude and oncological disease control. DFS could have helped determine whether extended resections provided any recurrence-related benefit, even if such a benefit did not translate into improved OS. Conversely, if DFS were also inferior in the extended resection group, this would further support the interpretation that these patients had more aggressive tumor biology rather than insufficient surgical extent. Because OS may be influenced by non-cancer-related deaths, postoperative physiological reserve, comorbidities, adjuvant treatment exposure, and subsequent therapies, the present findings should be interpreted as OS-based associations rather than definitive evidence of the oncological superiority or inferiority of a given surgical extent. Second, comprehensive comorbidity-adjusted analyses could not be performed reliably. Although we attempted to obtain detailed comorbidity profiles from institutional medical records, several older records were either not available in the digital archive or lacked standardized documentation of clinically relevant comorbid conditions. As a result, accurate calculation of a validated comorbidity index, such as the Charlson Comorbidity Index, was not feasible for the entire cohort. We acknowledge that this limits the ability to fully account for competing non-oncological causes of death, particularly because overall survival was used as the primary endpoint. Third, although all patients were routinely referred for multidisciplinary oncological evaluation, complete and verifiable data regarding the exact administration and completion rates of adjuvant chemotherapy were unavailable. Consequently, the potential impact of this treatment on survival outcomes could not be incorporated into our analysis. Finally, there is an inherent selection bias regarding the extent of surgical resection. It is crucial to note that the decision to perform extended resections was primarily dictated by the local invasiveness, size, and central location of the primary tumor, rather than by the extent of lymph node metastasis alone. Therefore, the diminished survival observed in the extended resection group cannot be attributed solely to nodal disease; it is likely confounded by the aggressive primary tumor characteristics that necessitated a more extensive surgical approach in the first place.

## 5. Conclusions

In conclusion, our findings underscore the necessity of subclassifying pN1 disease in future TNM staging systems. The anatomical location of N1 metastasis is a critical determinant of patient prognosis. We propose that an anatomical subclassification—defining N1a as strictly peripheral metastasis (stations 12–14) and N1b as hilar/interlobar metastasis (stations 10–11)—provides a more accurate prognostic stratification than numerical station counts. Standard lobectomy appeared to provide sufficient oncological outcomes in this cohort, and extended resections were not associated with improved overall survival. However, survival in pN1 disease is influenced not only by nodal anatomy but also by tumor-related biological factors, including T-stage, local invasiveness, and PNI. In this context, extended resections did not appear to improve survival and should not be routinely justified solely by proximal N1 involvement. Thus, our findings support the concept that proximal N1 involvement alone should not justify surgical escalation beyond the extent required to achieve complete primary tumor clearance.

Moreover, the absence of detailed data on adjuvant treatments in our cohort highlights the need for future studies to better define the relative contributions of surgical and systemic therapies within an individualized treatment framework.

## Figures and Tables

**Table 1:** Descriptive baseline clinicopathological characteristics of the overall cohort according to survival status.

	Survival Status		*p*-Value
	Non-Survivor (n = 72; 48%)	Survivor (n = 78; 52%)	
Gender, n (%)			0.583 ^a^
Female	11 (15.3)	12 (15.4)	
Male	61 (84.7)	66 (84.6)	
Age, years, Mean ± SD	63.35 ± 8.63	60.22 ± 8.22	0.051 ^b^
Median (Min–Max)	63.00 (38.00–82.00)	62.00 (38.00–76.00)	
T descriptor, n (%)			0.076 ^c^
T1b	2 (2.8)	3 (3.8)	
T1c	3 (4.2)	14 (17.9)	
T2a	14 (19.4)	14 (17.9)	
T2b	14 (19.4)	16 (20.5)	
T3	20 (27.8)	20 (25.6)	
T4	19 (26.4)	11 (14.1)	
TNM stage, n (%)			**0.026 ^c^**
IIA	5 (6.9)	17 (21.8)	
IIB	28 (38.9)	30 (38.5)	
IIIA	39 (54.2)	31 (39.7)	
Tumor histology, n (%)			0.411 ^c^
Adenocarcinoma	27 (37.5)	29 (37.2)	
Squamous cell carcinoma	36 (50.0)	44 (56.4)	
Others (mixed, large cell carcinoma, etc.)	9 (12.5)	5 (6.4)	
Operation type, n (%)			0.140 ^c^
Pneumonectomy	18 (25.0)	16 (20.5)	
Sleeve resection	2 (2.8)	8 (10.3)	
Bilobectomy	8 (11.1)	4 (5.1)	
Lobectomy	44 (61.1)	50 (64.1)	
Operation side, n (%)			0.211 ^a^
Right	34 (47.2)	43 (55.1)	
Left	38 (52.8)	35 (44.9)	
Lobe location, n (%)			0.691 ^c^
Middle or involving multiple lobes	19 (26.4)	16 (20.5)	
Upper	31 (43.1)	37 (47.4)	
Lower	22 (30.6)	25 (32.1)	
pN1 positive station(s), n (%)			0.356 ^c^
10	2 (2.8)	4 (5.1)	
10, 11	1 (1.4)	-	
10, 11, 12	1 (1.4)	1 (1.3)	
10, 12	2 (2.8)	6 (7.7)	
10, 12, 13–14	4 (5.6)	1 (1.3)	
11	6 (8.3)	7 (9.0)	
11, 12	3 (4.2)	1 (1.3)	
11, 12, 13–14	2 (2.8)	-	
12	36 (50.0)	39 (50.0)	
12, 13–14	5 (6.9)	9 (11.5)	
13–14	10 (13.9)	10 (12.8)	
Number of positive lymph nodes,			0.407 ^b^
Mean ± SD	2.39 ± 2.53	2.29 ± 2.27	
Median (Min–Max)	2.00 (1.00–19.00)	1.00 (1.00–16.00)	
Multiple station pN1, n (%)	17 (23.6)	18 (23.1)	0.546 ^a^
Lymphovascular invasion, n (%)	54 (75.0)	54 (69.2)	0.273 ^a^
Perineural invasion, n (%)	21 (29.2)	19 (24.4)	0.315 ^a^
Visceral pleura invasion, n (%)	34 (47.2)	24 (30.8)	**0.029 ^a^**
Surgical approach, n (%)			0.205 ^a^
VATS	23 (31.9)	31 (39.7)	
Thoracotomy	49 (68.1)	47 (60.3)	
Follow-up period (survival time)			
Mean ± SD	38.58 ± 34.03	95.87 ± 42.00	**0.000 ^b^**
Median (Min–Max)	28.50 (2.00–145.00)	98.50 (26.00–165.00)	

^a^. Fisher’s Exact Test, ^b^. Mann–Whitney U Test, ^c^. Chi-Square, SD: Standard Deviation. Bold values indicate statistically significant differences (*p* < 0.05).

**Table 2:** Spearman’s rank correlation analysis between clinicopathological parameters and survival duration.

Survival Time	Correlation Coefficient (r)	*p*-Value
Gender	0.011	0.897
**Age**	−0.237 **	**0.004**
**T descriptor**	−0.253 **	**0.002**
**TNM stage**	−0.213 **	**0.009**
Tumor histology	−0.067	0.413
Operation type	−0.001	0.989
**Operation side**	−0.190 *	**0.020**
Lobe location	−0.006	0.941
Number of positive pN1	−0.121	0.141
Multiple station pN1	−0.056	0.496
Lymphovascular invasion	−0.112	0.171
**Perineural invasion**	−0.164 *	**0.045**
**Visceral pleura invasion**	−0.257 **	**0.001**
Surgical approach (VATS vs. Thoracotomy)	−0.032	0.697
Hilar (10) pN1 positivity	−0.070	0.394
**Interlobar (11) pN1 positivity**	−0.220 **	**0.007**
Lobar (12) pN1 positivity	0.036	0.665

* *p* < 0.05, ** *p* < 0.01, bold values indicate significant correlation. r: Spearman’s correlation coefficient.

**Table 3:** Multivariable Cox proportional hazards regression analysis of independent predictors for overall survival.

	*p*-Value	Hazard Ratio-HR	95.0% CI for HR	
			Lower	Upper
**Age**	**0.026**	1.036	1.004	1.068
T descriptor	0.337	1.254	0.790	1.992
TNM stage	0.879	0.929	0.361	2.393
Perineural invasion	0.266	1.341	0.799	2.252
Visceral pleura invasion	0.062	1.604	0.977	2.632
**Interlobar (11) pN1 positivity**	0.044	1.912	1.018	3.593

CI: Confidence Interval. Bold values indicate statistically significant differences (*p* < 0.05).

**Table 4:** Baseline Characteristics According to Anatomical pN1 Location.

	Extent of Lymph Node Positivity		*p*-Value
	Peripheral pN1 (Stations 12–14)	Hilar/Interlobar pN1 (Stations 10–11)	
Gender, n (%)			0.055 ^a^
Female	13 (11.9)	10 (24.4)	
Male	96 (88.1)	31 (75.6)	
Age, years, Mean ± SD	61.34 ± 8.87	62.73 ± 7.61	0.609 ^b^
Median (Min–Max)	62.00 (38.00–82.00)	63.00 (45.00–76.00)	
T descriptor, n (%)			0.671 ^c^
T1b	3 (2.8)	2 (4.9)	
T1c	11 (10.1)	6 (14.6)	
T2a	22 (20.2)	6 (14.6)	
T2b	20 (18.3)	10 (24.4)	
T3	32 (29.4)	8 (19.5)	
T4	21 (19.3)	9 (22.0)	
TNM stage, n (%)			0.540 ^c^
IIA	14 (12.8)	8 (19.5)	
IIB	42 (38.5)	16 (39.0)	
IIIA	53 (48.6)	17 (41.5)	
**Tumor histology, n (%)**			**0.010 ^c^**
**Adenocarcinoma**	**38 (34.9)**	**18 (43.9)**	
**Squamous cell carcinoma**	**65 (59.6)**	**15 (36.6)**	
**Others (mixed, large cell carcinoma, etc.)**	**6 (5.5)**	**8 (19.5)**	
Operation type, n (%)			0.983 ^c^
Pneumonectomy	24 (22.0)	10 (24.4)	
Sleeve resection	7 (6.4)	3 (7.3)	
Bilobectomy	9 (8.3)	3 (7.3)	
Lobectomy	69 (63.3)	25 (61.0)	
Operation side, n (%)			0.566 ^a^
Right	56 (51.4)	21 (51.2)	
Left	53 (48.6)	20 (48.8)	
Lobe location, n (%)			0.075 ^c^
Middle or involving multiple lobes	25 (22.9)	10 (24.4)	
Upper	55 (50.5)	13 (31.7)	
Lower	29 (26.6)	18 (43.9)	
pN1 positive station(s), n (%)			0.000 ^c^
10	-	6 (14.6)	
10, 11	-	1 (2.4)	
10, 11, 12	-	2 (4.9)	
10, 12	-	8 (19.5)	
10, 12, 13–14	-	5 (12.2)	
11	-	13 (31.7)	
11, 12	-	4 (9.8)	
11, 12, 13–14	-	2 (4.9)	
12	75 (68.8)	-	
12, 13–14	14 (12.8)	-	
13–14	20 (18.3)	-	
Number of positive lymph nodes, Mean ± SD	2.11 ± 1.97	2.95 ± 3.20	0.337 ^b^
Median (Min–Max)	1.00 (1.00–16.00)	2.00 (1.00–19.00)	
**Multiple station pN1, n (%)**	**13 (11.9)**	**22 (53.7)**	**0.000 ^a^**
Lymphovascular invasion, n (%)	78 (71.6)	30 (73.2)	0.509 ^a^
Perineural invasion, n (%)	28 (25.7)	12 (29.3)	0.402 ^a^
Visceral pleura invasion, n (%)	41 (37.6)	17 (41.5)	0.402 ^a^
Surgical approach, n (%)			0.536 ^a^
VATS	39 (35.8)	15 (36.6)	
Thoracotomy	70 (64.2)	26 (63.4)	

^a^. Fisher’s Exact Test, ^b^. Mann Whitney U Test, ^c^. Chi-Square, SD: Standard Deviation. Bold values indicate statistically significant differences (*p* < 0.05).

**Table 5:** Comparison of survival times according to the anatomical level of pN1 lymph node metastasis.

Survival Time			*p*-Value
Positive N1 Stations	Mean ± SD	Median (Min–Max)	
Stations 12–14	73.61 ± 47.79	66.00 (2.00–165.00)	0.019 ^a^
Stations 10–11	54.44 ± 45.65	41.00 (2.00–155.00)	

^a^. Mann–Whitney U Test. SD: Standard deviation; Min: Minimum; Max: Maximum.

**Table 6:** Impact of surgical extent (standard lobectomy vs. extended resections) on survival in hilar/interlobar and peripheral pN1 disease.

Extent of Resection	Survival Time (Months)		
	Mean ± Std. Deviation	Median (Min–Max)	*p*-Value
**Peripheral pN1 (Stations 12–14)**			0.246 ^a^
Extended resections	81.80 ± 52.26	71.50 (9.00–164.00)	
Standard lobectomy	68.87 ± 44.70	60.00 (2.00–165.00)	
**Hilar/Interlobar pN1 (Stations 10–11)**			0.046 ^a^
Extended resections	39.81 ± 40.48	28.50 (2.00–131.00)	
Standard lobectomy	63.80 ± 47.06	50.00 (6.00–155.00)	

^a^. Mann–Whitney U test. SD: Standard deviation; Min: Minimum; Max: Maximum.

**Table 7:** Impact of primary tumor characteristics and multiple-station N1 disease on survival in Stage II and Stage III patients.

Probable Risk Factor for Survival		TNM Stage			
		Stage II (IIA–IIB)		Stage III (IIIA)	
		Survival Time (Months)	*p*-Value	Survival Time (Months)	*p*-Value
		Mean ± SD		Mean ± SD	
N1 station involvement	Single station	78.55 ± 45.70	0.438 ^a^	58.08 ± 47.09	0.879 ^a^
	Multiple station	69.00 ± 50.99		61.21 ± 50.82	
Lymphovascular invasion	No	79.73 ± 51.13	0.742 ^a^	76.30 ± 56.72	0.111 ^a^
	Yes	75.47 ± 45.22		51.98 ± 42.36	
**Perineural invasion**	No	77.49 ± 45.29	0.694 ^a^	**66.67 ± 49.51**	**0.027 ^a^**
	Yes	74.24 ± 51.28		**38.16 ± 36.30**	
Visceral pleura invasion	No	80.37 ± 46.35	0.113 ^a^	70.15 ± 48.59	0.078 ^a^
	Yes	60.47 ± 45.83		51.88 ± 46.45	

^a^. Mann–Whitney U Test. SD: Standard deviation. Bold values indicate statistically significant differences (*p* < 0.05).

## Data Availability

The raw data supporting the conclusions of this article will be made available by the authors on request.
